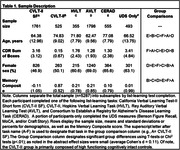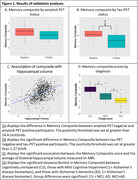# A harmonized memory composite score for cross‐cohort Alzheimer’s disease and related dementia research: development and validation

**DOI:** 10.1002/alz.093383

**Published:** 2025-01-03

**Authors:** Mark E. Sanderson‐Cimino, Alden L. Gross, Leslie S. Gaynor, Emily W. Paolillo, Kaitlin B. Casaletto, Ankita Chatterjee, Marilyn S. Albert, Liana G. Apostolova, Brooke Boersema, Adam L. Boxer, Brad F. Boeve, Lindsay R. Clark, Renaud La Joie, Ani Eloyan, Sarah Tomaszewski Farias, Mitzi M. Gonzales, Dustin B. Hammers, Amy B. Wise, Yann Cobigo, Claire Yballa, Daniel R. Schonhaut, Benjamin M. Hampstead, Dawn Mechanic‐Hamilton, Bruce L. Miller, Gil D. Rabinovici, Katya Rascovsky, John M Ringman, Howard J. Rosen, Sephira Ryman, David P. Salmon, Glenn E. Smith, Charles Decarli, Joel H. Kramer, Adam M. Staffaroni

**Affiliations:** ^1^ Memory and Aging Center, UCSF Weill Institute for Neurosciences, University of California, San Francisco, San Francisco, CA USA; ^2^ Center on Aging and Health, Johns Hopkins University, Baltimore, MD USA; ^3^ University of California, San Francisco, San Francisco, CA USA; ^4^ Johns Hopkins University School of Medicine, Baltimore, MD USA; ^5^ Indiana Alzheimer’s Disease Research Center, Indianapolis, IN USA; ^6^ Indiana University, Indianapolis, IN USA; ^7^ Mayo Clinic, Rochester, MN USA; ^8^ University of Wisconsin‐Madison School of Medicine and Public Health, Madison, WI USA; ^9^ Memory and Aging Center, Weill Institute for Neurosciences, University of California, San Francisco, San Francisco, CA USA; ^10^ Brown University, Providence, RI USA; ^11^ University of California, Davis, Sacramento, CA USA; ^12^ Glenn Biggs Institute for Alzheimer’s & Neurodegenerative Diseases, University of Texas Health Science Center, San Antonio, TX USA; ^13^ Indiana University School of Medicine, Indianapolis, IN USA; ^14^ Weill Institute for Neurosciences, University of California, San Francisco (UCSF), San Francisco, CA USA; ^15^ University of Michigan, Ann Arbor, MI USA; ^16^ University of Pennsylvania, Philadelphia, PA USA; ^17^ University of Southern California, Los Angeles, CA USA; ^18^ Memory and Aging Center, UCSF Weill Institute for Neurosciences, San Francisco, CA USA; ^19^ Mind Research Network, Albuquerque, NM USA; ^20^ University of California, San Diego, La Jolla, CA USA; ^21^ University of Florida, Gainesville, FL USA; ^22^ University of California, Davis, Davis, CA USA

## Abstract

**Background:**

The Uniform Data Set (UDS) neuropsychological battery, administered across Alzheimer’s Disease Centers (ADC), includes memory tests but lacks a list‐learning paradigm. ADCs often supplement the UDS with their own preferred list‐learning task. Given the importance of list‐learning for characterizing memory, we aimed to develop a harmonized memory score that incorporates UDS memory tests while allowing centers to contribute differing list‐learning tasks.

**Method:**

We applied item‐banking confirmatory factor analysis to develop a composite memory score in 5,287 participants (mean age 67.1; SD = 12.2) recruited through 18 ADCs and four consortia (DiverseVCID, MarkVCID, ALLFTD, LEADS) who completed UDS memory tasks (used as linking‐items) and one of five list‐learning tasks. All analyses used linear regression. We tested whether memory scores were affected by which list‐learning task was administered. To assess construct validity, we tested associations of memory scores with demographics, disease severity (CDR Box Score), an independent memory task (TabCAT Favorites, n = 675), and hippocampal volume (n = 811). We compared performances between cognitively unimpaired (n = 279), AD‐biomarker+ MCI (n = 26), and AD‐biomarker+ dementia (n = 98). In a subsample with amyloid‐ and tau‐PET (n = 49), we compared memory scores from participants with positive vs negative scans determined using established quantitative cutoffs.

**Result:**

Model fit indices were excellent (e.g., CFI = 0.998) and factor loadings were strong (0.43‐0.93). Differences in list‐learning task had a negligible effect on scores (average Cohen’s d = 0.11). Higher memory scores were significantly (*p*’s<.001) correlated with younger age (β = ‐0.18), lower CDR Box Scores (β = ‐0.63), female sex (β = 0.12), higher education (β = 0.19), larger hippocampal volume (β = 0.42), and an independent memory task (β = 0.71, p<0.001). The memory composite declined in a stepwise fashion by diagnosis (cognitively unimpaired>MCI>AD dementia, p<0.001). On average, amyloid‐PET positivity was associated with lower composite scores, but was not statistically significant (β = ‐0.34; p = 0.25; d = 0.40). Tau‐PET positivity was associated with worse performance, demonstrating a large effect size (β = ‐0.75; p<0.002; d = 0.91).

**Conclusion:**

The harmonized memory score developed in a large national sample was stable regardless of contributing list‐learning task and its validity for cross‐cohort ADRD research is supported by expected associations with demographics, clinical measures, and Alzheimer’s biomarkers. A processing script will be made available to enhance cross‐cohort ADRD research.